# Transcriptome wide analyses reveal a sustained cellular stress response in the gill tissue of *Trematomus bernacchii* after acclimation to multiple stressors

**DOI:** 10.1186/s12864-016-2454-3

**Published:** 2016-02-20

**Authors:** Troy J. Huth, Sean P. Place

**Affiliations:** Department of Biology, Sonoma State University, Rohnert Park, CA 94928 USA; Department of Biological Sciences, University of South Carolina, Columbia, SC 29208 USA

**Keywords:** Ocean acidification, Thermal stress, RNA-seq, Notothenioid, Gene expression

## Abstract

**Background:**

As global climate change progresses, the Southern Ocean surrounding Antarctica is poised to undergo potentially rapid and substantial changes in temperature and *p*CO_2._ To survive in this challenging environment, the highly cold adapted endemic fauna of these waters must demonstrate sufficient plasticity to accommodate these changing conditions or face inexorable decline. Previous studies of notothenioids have focused upon the short-term response to heat stress; and more recently the longer-term physiological response to the combined stress of increasing temperatures and *p*CO_2_. This inquiry explores the transcriptomic response of *Trematomus bernacchii* to increased temperatures and *p*CO_2_ at 7, 28 and 56 days, in an attempt to discern the innate plasticity of *T. bernacchii* available to cope with a changing Southern Ocean.

**Results:**

Differential gene expression analysis supported previous research in that *T. bernacchii* exhibits no inducible heat shock response to stress conditions. However, *T. bernacchii* did demonstrate a strong stress response to the multi-stressor condition in the form of metabolic shifts, DNA damage repair, immune system processes, and activation of apoptotic pathways combined with negative regulation of cell proliferation. This response declined in magnitude over time, but aspects of this response remained detectable throughout the acclimation period.

**Conclusions:**

When exposed to the multi-stressor condition, *T. bernacchii* demonstrates a cellular stress response that persists for a minimum of 7 days before returning to near basal levels of expression at longer acclimation times. However, subtle changes in expression persist in fish acclimated for 56 days that may significantly affect the fitness *T. bernacchii* over time.

**Electronic supplementary material:**

The online version of this article (doi:10.1186/s12864-016-2454-3) contains supplementary material, which is available to authorized users.

## Background

Isolation of the Antarctic continental shelf by the Polar Front has arguably produced the coldest, most oceanographically stable environment on the planet. However, this long-term oceanographic stability may have resulted in the evolution of an ecosystem filled with endemic fauna that are poorly poised to deal with rapid climate variation [1, 2]. In the face of global climate change, marine organisms are perceived to have but three options: they can migrate to more favorable environments, alter their biology through physiological plasticity, or evolve in response to the altered environment [3–7]. Given the unique environment afforded by the Southern Ocean, it is highly unlikely that population migration is a viable option for its endemic biota. While evolutionary responses may benefit the species in some longer time frame, they may be outpaced by environmental change. Thus, for extant communities in the Antarctic, use of physiologically plastic responses may be the only available method of persisting under near-term future environmental variations.

The Southern Ocean is dominated by an endemic suborder of perciform fishes, the Notothenioidei [8]. The effects of increased temperature on the physiology of Antarctic fish have been well documented providing some important insight into the plasticity of a number of Antarctic fish species to a single stress [9–16]. However, we have very little information regarding what impact interacting and synergistic stressors will have on the physiological tolerances of these unique fish. This is problematic for identifying physiological tipping-points for polar species, as previous measurements of their capacity to respond may be overly conservative since they do not account for non-additive effects of the joint action of multiple stressors. This point has been highlighted by recent studies that assessed the physiological response of temperate and eurythermal organisms to the combined stress of ocean acidification and elevated temperature. Results reported from these studies suggest significant trade-offs in performance and stress tolerance may exist when two or more environmental conditions are varied [17–19]. An important outcome of these studies is an increased understanding among physiological ecologists that relevant assessment of species vulnerabilities will require consideration of multiple environmental variables [20, 21]. Equally important is the realization that it is no longer sufficient to focus on a single cellular or molecular response, but rather the consideration of multiple co-regulated processes is critical [20, 22].

Genomics-based approaches hold the promise of greatly facilitating our understanding of physiological plasticity in these endemic fishes, especially when considering the impact dynamic environments have on multiple physiological pathways [23–26]. With modern genomic techniques, we can now ask: to what extent are conserved patterns of gene expression absent in the Antarctic fishes and how does this affect their ability to adjust to major changes in their environment? To this end, we have used RNA-seq analyses to profile the genomic response of an endemic Antarctic fish to predicted levels of ocean acidification and global increases in mean sea surface temperature (SST). In this investigation of multiple stressors related to climate change, our goal was to assess the molecular response of *Trematomus bernacchii* to conditions consistent with scenarios laid out by the IPCC with respect to anthropogenic increases in atmospheric CO_2_ [6] while providing direct comparison to previous studies looking at thermal stress in this organism.

## Results and Discussion

### Reference transcriptome

Following assembly, the transcriptomic library initially consisted of 421,044 unigenes (unique gene products including all isoforms) and 537,064 transcripts with a median transcript length of 444 bp, mean transcript length of 1011 bp and N50 of 2160 bp. After removing transcripts with expression levels below 0.001 FPKM (fragments per kilobase million) and clustering at 100 % identity; 314,638 transcripts and 246,333 unigenes remained. Transcript level annotation yielded 98,451 BLAST hits (1 × 10e^−6^ cutoff value) and 34,096 GO (gene ontology) annotations.

### Sequencing read quality control and mapping

Sequencing yielded an average of 27,141,939 reads (s.d. = ±2,544,475; min = 22,080,886; max = 30,834,415) per sample. Trimmomatic [27] quality processing retained an average of 94.28 % (s.d. = ±0.14 %; min = 93.99 %; max = 94.58 %) of the input reads; resulting in samples containing an average of 25,591,193 reads (s.d. = ±2,413,885; min = 20,760,581; max = 29,083,777). Bowtie2 [28] mapping achieved an average of 90.90 % (s.d. = ±5.76 %; min = 89.52 %; max = 91.72 %) of the trimmed sequencing reads mapped to the reference transcriptome, which corresponded to an average number of mapped reads per sample of 23,261,884 (s.d. = ±2,206,537; min = 18,817,366; max = 26,562,213).

### Transcriptome-wide differential gene expression analysis

**Fig. 1:**
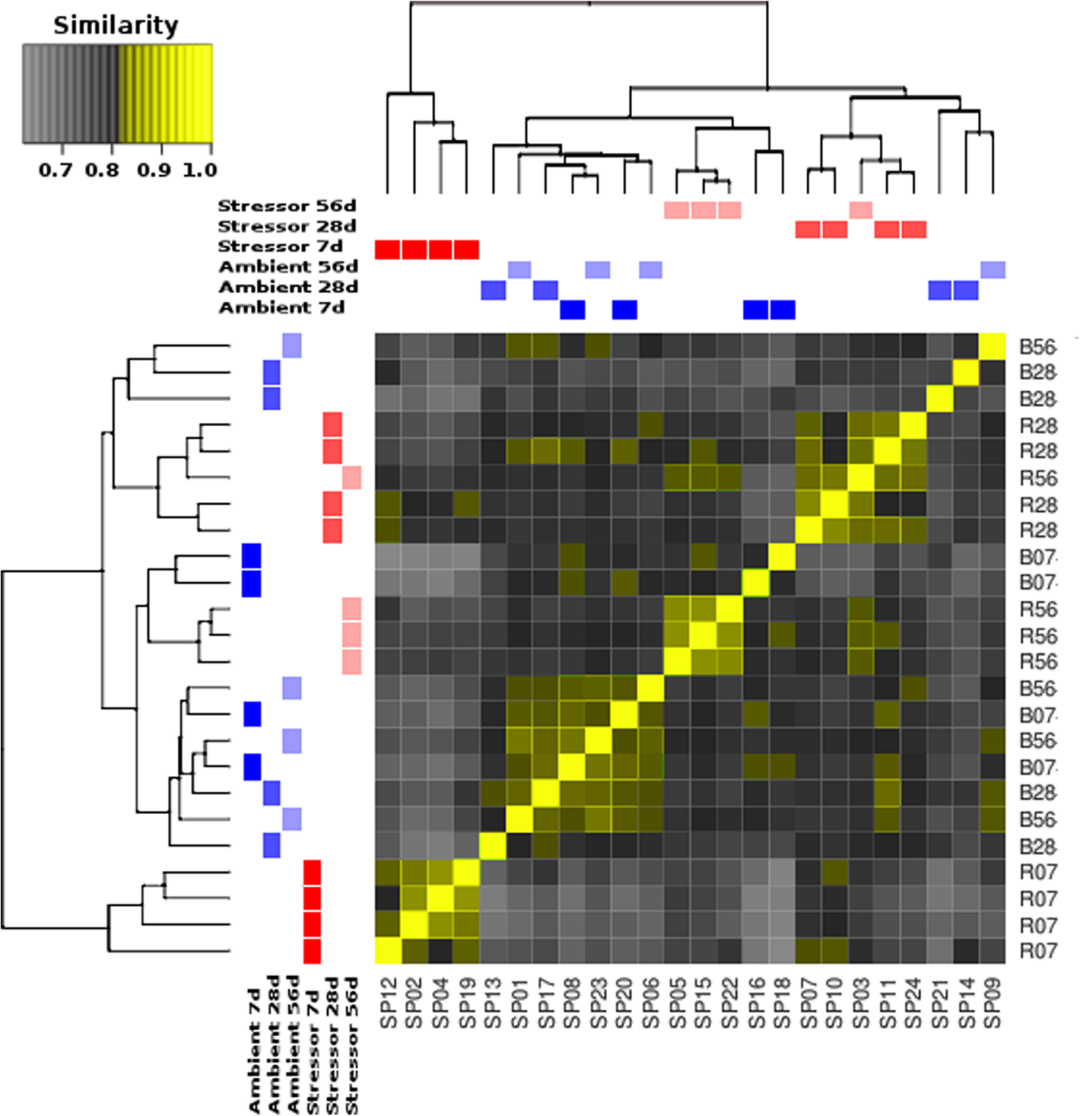
Transcriptome wide sample similarity matrix and cluster dendrogram: the sample similarity matrix represents the cumulative similarity of each individual at each time-point as a reflection of transcriptome wide gene expression. Transcriptome wide expression is represented by all gene products that demonstrated an FDR ≤ 0.05 during the differential gene expression analysis. Portions of the matrix shown in *yellow* demonstrate a high degree of similarity in the transcriptomic expression profiles between the two samples, with a value of 1 indicating the samples are identical; whereas those shown in *grey* demonstrate a lower degree of similarity, with a value of 0.65 demonstrating the most dissimilar expression profiles between two samples. As the samples are extracted from the same tissue and species, the degree of similarity in this case is a minimum of 0.65. The cluster dendrogram also groups samples based upon similar expression profiles, with those samples grouped most closely demonstrating more analogous transcriptomic expression responses

Differential gene expression analysis using edgeR [29] yielded a total of 4880 differentially expressed genes across all three time points (FDR ≤ 0.05). A sample similarity comparison demonstrated that the 7d multi-stressor treatment resulted in considerable differential gene expression when compared to the 7d control treatment (Fig. 1). Furthermore, the 7d multi-stressor individuals clustered as an outgroup in the cluster dendrogram of all treatments and time-points, demonstrating the consistent and distinct effect of this treatment-time combination on overall gene expression as compared to all others (Fig. 1). The 28d and 56d multi-stressor treatments also segregated from their respective control treatments indicating differential expression compared to the control, although to a lesser extent than the 7d multi-stressor treatment (Fig. 1).

**Fig. 2:**
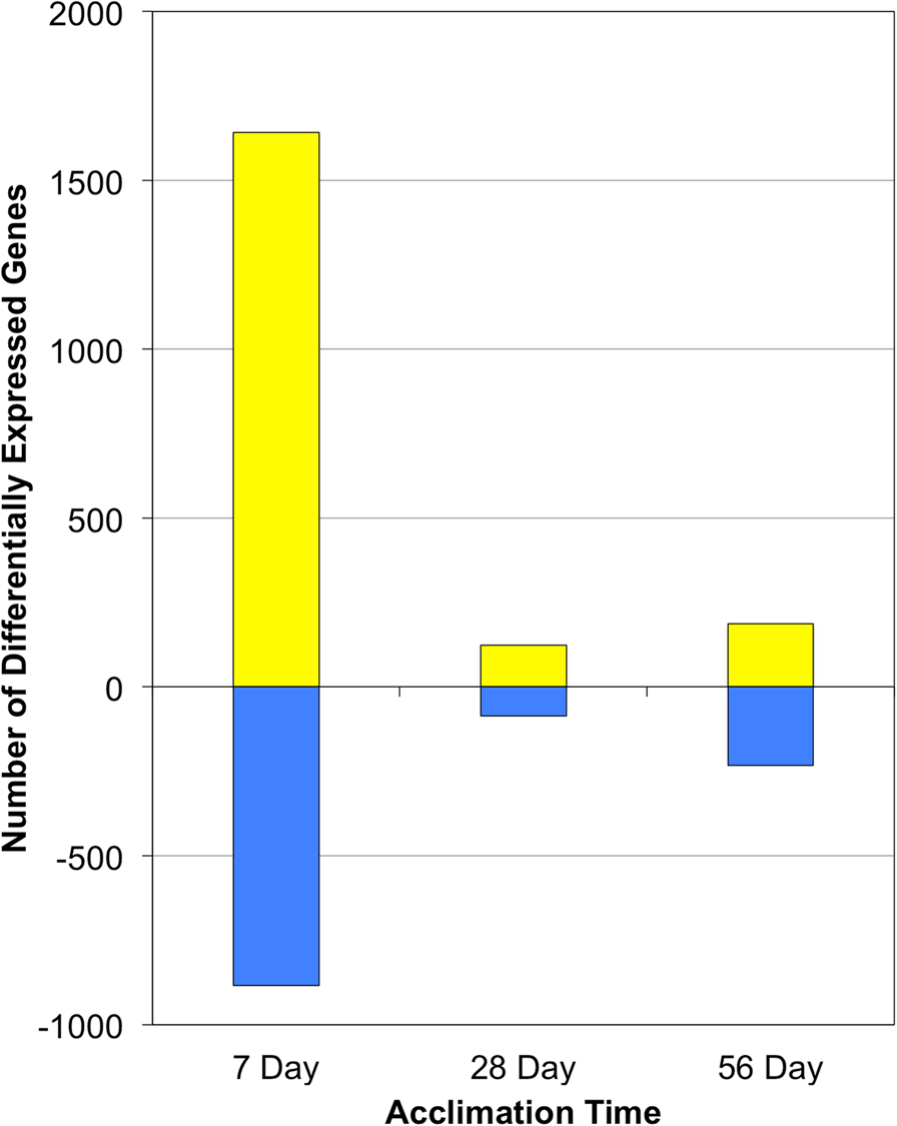
Number of differentially expressed genes: the bar graph depicts the total number of differentially expressed genes observed between the multi-stressor treatment and control fish at each time point. *Yellow bars* (positive values) represent the total number of DE genes up-regulated after 7, 28, & 56 days of acclimation while *blue bars* (negative values) represent the total number of DE genes down-regulated. In all, differential gene expression analysis using edgeR [29] yielded a total of 4880 differentially expressed genes across all three time points (FDR ≤ 0.05). Fish from the 7d multi-stressor treatment displayed 1642 up-regulated and 886 down-regulated. Fish from the 28d multi-stressor treatment displayed 123 up-regulated and 86 down-regulated. Fish from the 56d multi-stressor treatment displayed 187 up-regulated and 232 down-regulated

Direct comparisons of the control and multi-stressor treatment at each time-point demonstrates 2528 differentially expressed genes within the 7d multi-stressor treatment; 209 differentially expressed genes within the 28d multi-stressor treatment; and 419 differentially expressed genes within the 56d multi-stressor treatment. Of the 2528 differentially expressed genes of the 7d multi-stressor treatment, 1642 were up-regulated and 886 were down-regulated (Fig. 2). Of the 209 differentially expressed genes in the 28d multi-stressor treatment 123 were up-regulated with 86 down-regulated (Fig. 2). Lastly, the 56d multi-stressor treatment demonstrated 187 up-regulated genes and 232 down-regulated genes out of the 419 total differentially expressed (Fig. 2). These metrics indicate a robust initial response to the 7d multi-stressor treatment, which tapers off considerably in fish acclimated to the multi-stressor treatment for 28d and 56d (Fig. 2). A slight increase in the number of differentially expressed genes was observed between the 28d and 56d time-points which may provide insight into the long-term acclimation tactics in these fish (Fig. 2).

### Gene ontology over-representation analysis

Fisher’s Exact Tests for gene ontology term over-representation further supported a robust initial transcriptome-wide response in gill tissues that diminishes over time, with the 7d stressor treatment exhibiting the most differentially expressed and over-represented gene ontology terms.

**Fig. 3:**
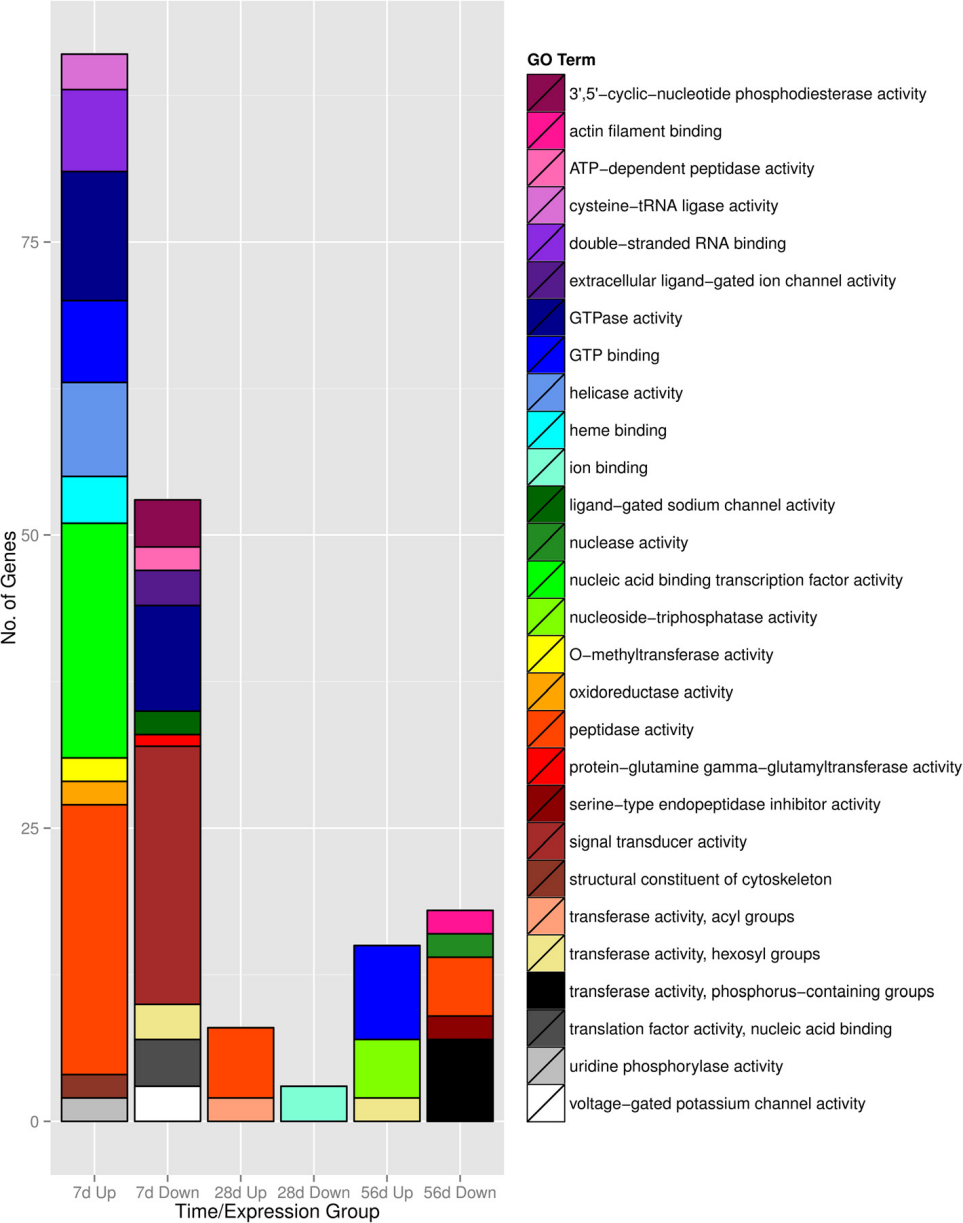
Over-representation analysis of gene ontology level 2 category molecular function: shown are gene ontology terms within the broad molecular function category (GO:0003674) that were significantly over-represented in the stressor treatments compared to the reference transcriptome as determined by a Fisher’s exact test (*p* < 0.01, categories containing only one gene are not included for readability). Subgroups of up- and down-regulated gene cohorts were created for each stressor time-point (7d, 28d and 56d) and compared to the GO term distribution of the reference transcriptome. The number in each category represents the total number of genes within that over-expressed GO term category (blank = 0)

For fish within the 7d multi-stress acclimation group, GO terms within the molecular function subclasses associated with differentially up-regulated genes indicate these fish experienced large-scale cellular remodeling (Fig. 3, Additional file 1: Table S1). Among the up-regulated genes in the 7d multi-stressor treatment we found that GO terms associated with nucleic acid binding, transcription factor activity, helicase activity, and double-stranded RNA binding activity were over-represented; indicating a significant change in transcription activation and RNA processing. Furthermore, the increased expression of GTPase activity, GTP binding, and tRNA ligase activity suggests a significant up-regulation of a diverse number of cellular functions such as trafficking across the nuclear membrane and protein biosynthesis in the 7d multi-stressor-acclimated group. Lastly, there also appears to be a considerable amount of protein recycling occurring in fish acclimated to the multi-stress treatment for 7 days as evidence by the significant increase in the expression of 23 genes associated with peptidase activity. This spike in protein degradation coincides with a marked increase in oxidative damaged in the same fish tissue [30]. It has been previously demonstrated that transcriptional regulation, RNA processing, protein biosynthesis and proteolysis are highly active gene ontology groups when exposing *T. bernacchii* to short term heat stress (4 °C for 4 h) [31]; our findings indicate that this initial cellular stress response continues well into the first 7 days. It was further observed that peptidase activity remained up-regulated in fish acclimated to the multi-stressor treatment for 28d, before being down-regulated in the 56d stressor treatment specimens.

**Fig. 4:**
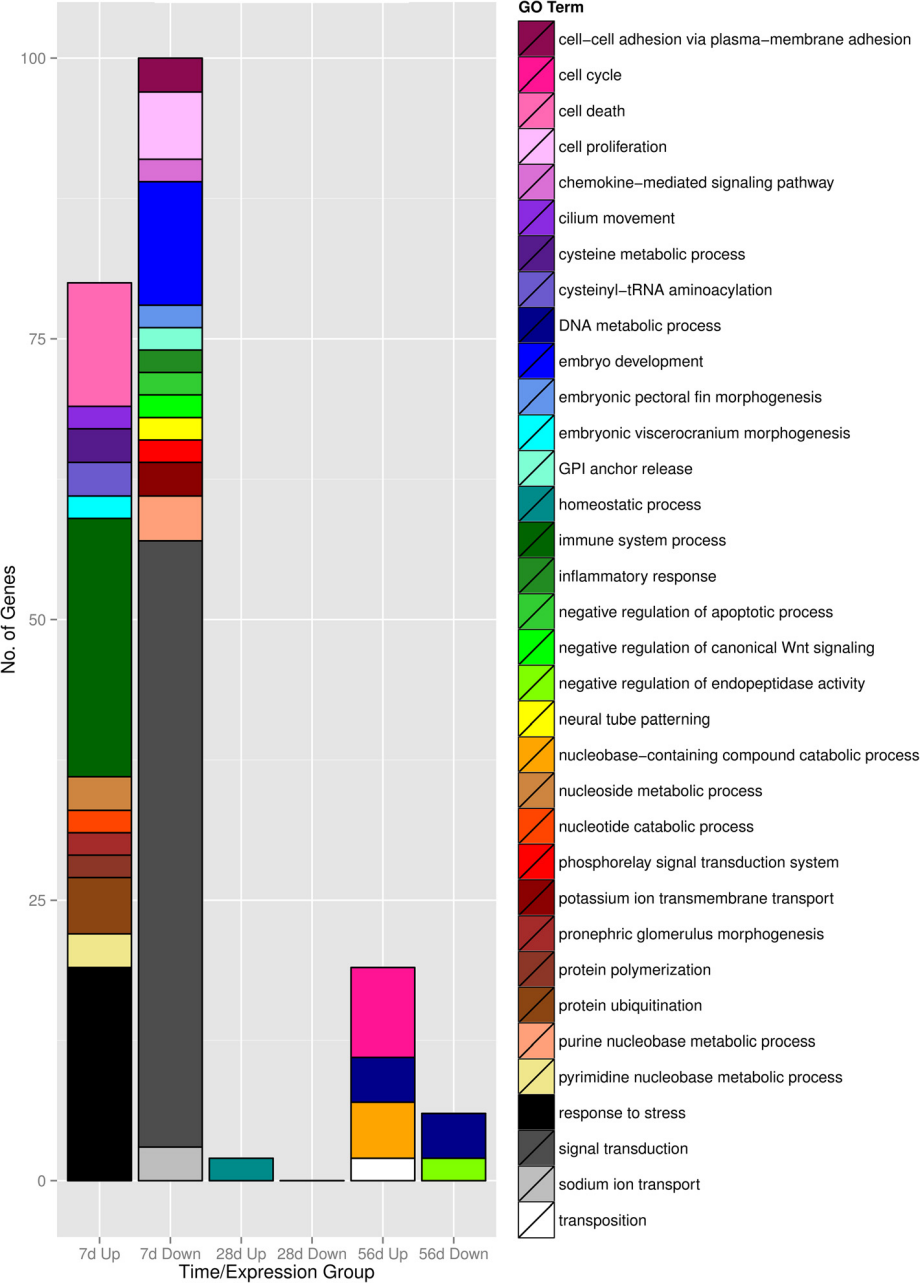
Over-representation analysis of the gene ontology level 2 category biological process: gene ontology terms within the broad biological process category (GO:0008150) that were significantly over-represented in the stressor treatments compared to the reference transcriptome as determined by a Fisher’s exact test (*p* < 0.01, categories containing only one gene are not included for readability). Subgroups of up- and down-regulated gene cohorts were created for each stressor time-point (7d, 28d and 56d) and compared to the GO term distribution of the reference transcriptome. The number in each category represents the total number of genes within that over-expressed GO term category (blank = 0)

Over-representation analysis of GO terms in the biological processes category identified a significant number of genes associated with a sustained response to cellular damage in the gill cells of fish acclimated to the 7d multi-stressor treatment (Fig. 4, Additional file 1: Table S1). The top 4 over-represented categories included immune system response (23 genes), response to stress (19), cell death (11), and protein ubiquitination (5). Whereas the biological processes highly over-represented in the down-regulated genes in the gill tissues of the same fish include signal transduction (54 genes), embryo or morphological development (11), and cell proliferation (6). The over-representation of any biological processes is largely absent in fish acclimated to the multi-stressor treatment for 28d, while we observed a slight increase in the positive regulation of the cell cycle (8) and DNA metabolic processes (5) in the 56d multi-stressor treatment. Taken together, the analysis of molecular and biological functions suggest *T. bernacchii* maintains a sustained response to cellular stress in its gills for at least 7 days and returns to a homeostatic state by 28 days of acclimation. Specimens acclimated to the multi-stressor treatment may experience a second adjustment in transcriptional activity that may indicate a potentially prolonged acclimation response at the 56d time-point.

Organisms experiencing environmental stress can often display two conserved responses, a rapid, transient response known as the cellular stress response (CSR) and a more permanent response termed the cellular homeostasis response (CHR) [32]. It is likely expression profiles observed in the 7d and then the 28d or 56d acclimated fish highlights the transition from the CSR to the CHR, and are representative of both the immediate and long-term adjustments necessary to cope with these environmental conditions. Therefore, a more detailed analysis of the biological processes found to be over-represented was conducted for cell death and immune system process; in addition to a number of more specific GO terms for biological processes potentially involved in the acclimation to multiple stressors including: carbohydrate metabolic processes, lipid metabolic processes, cell proliferation, cell death, response to stress, and homeostatic processes. These sub-categories are further investigated below in an attempt to identify specific gene products involved in the initial 7d time-point response, and those that may indicate a capacity for long term acclimation at the 28d and 56d time-points (Table 1).

**Table 1:** A summary of major gene ontology groups demonstrating differential gene expression at the 7d, 28d, and 56d time-points of the multi-stressor condition

Gene ontology category	Total genes found in the transcriptome	7d differentially expressed			28d differentially expressed			56d differentially expressed		
		Total	Up	Down	Total	Up	Down	Total	Up	Down
Carbohydrate metabolic process	480	61	30	31	2	2	0	9	8	1
Cell death	171	32	20	12	3	1	2	4	1	3
Cell proliferation	155	19	4	15	0	0	0	1	1	0
Homeostatic process	194	20	18	2	2	2	0	9	5	4
Immune system process	246	53	39	14	7	7	0	9	5	4
Lipid metabolic process	409	42	20	22	4	4	0	8	6	2
Response to stress	420	62	48	14	3	3	0	12	7	5

The first column includes the total number of genes found within the transcriptome associated with each GO term, followed by the differential expression data for each time-point of the multi-stressor treatments as compared to the control treatment (sub-divided into total, up- and down-regulated) for each of the GO term categories carbohydrate metabolic process, cell death, cell proliferation, homeostatic process, immune system process, lipid metabolic process, and response to stress. The confidence threshold to determine differential expression was a FDR ≤ 0.05

To highlight the molecular response of these fish to acute and chronic stress, we have separated our discussion into two primary sections consisting of the short-term responses (7d) and the longer-term responses (28 and 56d).

## Pathway specific responses (7d multi-stressor treatment)

### Carbohydrate and lipid metabolism

**Fig. 5:**
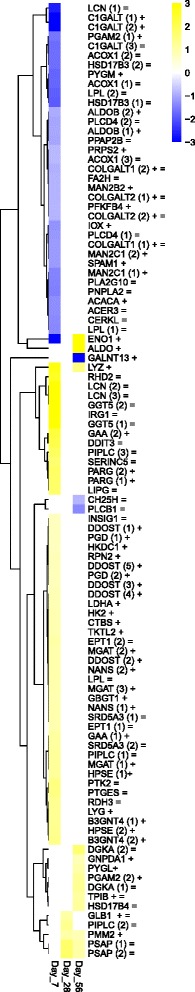
Heatmap–metabolic processes: this heatmap demonstrates the change in expression patterns of metabolic genes that displayed differential expression in at least one acclimation time point (7d, 28d, or 56d) when fish acclimated to the multi-stressor treatment (*n* = 4) were compared to fish in the control treatments (*n* = 4). Genes associated with the gene ontology term carbohydrate metabolic process (GO:0005975) are represented by (+) and lipid metabolic process (GO:0006629) are represented by (=) . Fold change is log_2_ adjusted with genes showing significant up-regulation depicted in *yellow* and genes showing a significant down-regulation in *blue* (FDR ≤ 0.05). Genes whose level of expression did not differ significantly between control and multi-stressor treatments are shown in *white*. The number of gene variants identified in the annotated transcriptome that were associated with the same gene name are indicated in *parentheses* next the gene symbol

Analysis of significant changes in mRNA expression levels for genes associated with the GO category for carbohydrate metabolism (Fig. 5, Additional file 2: Table S2) demonstrated a nearly equal number of genes that were up- and down-regulated at 7d in fish acclimated to the multi-stressor treatment relative to the control treatment (30 up-regulated, 31 down-regulated). Of the up-regulated genes in this category, nearly half (12) were associated with hydrolysis of carbohydrates while 13 genes displaying significant down-regulation were associated with carbohydrate synthesis. The same general trend was observed in lipid metabolism, with 20 genes up-regulated and 22 genes down-regulated in fish acclimated to the multi-stressor treatment for 7d. Of the genes found to be up-regulated at the 7d time-point, a large number of genes were associated with small molecule metabolic processes (11), most notably genes involved in phospholipid biosynthesis and membrane maintenance such as SERINC5, PLCXD1, PLCXD3, and EPT1. Among the down-regulated genes, 16 were associated with lipid biosynthesis or catabolism (Fig. 5, Additional file 3: Table S3).

Previous heat stress studies in Notothenioids have demonstrated an up-regulation of genes associated with both carbohydrate and lipid metabolism, albeit it on a much shorter time scale of 4 h [31]. An increased glycolytic capacity is paralleled by a significant increase in resting metabolic rates when specimens are acclimated to stressful conditions for 7 days [33]. The rapid increase in capacity and oxygen consumption likely fuels the massive cellular reorganization captured in the expression profiles of fish acclimated to the multi-stressor treatment for 7 days. We have more recent metabolic rate data that provides further support for this hypothesis. The metabolic rates of these fish were measured over the 56d acclimation period, and similar to the 2013 study [33], these fish showed a dramatic increase in resting metabolic rate (RMR) within the first 7d of acclimation to the multi-stressor treatment, however, RMR rates in these fish returned to basal levels by 56d [34].

Additionally, notothenioids are thought to rely primarily on lipids to fuel glycolytic metabolism under non-stressed conditions [12, 35]; however, the significant down-regulation of lipid mobilization and catabolism pathways observed after 7 days of acclimation to stressful conditions suggests *T. bernacchii* has shifted away from its reliance on lipids as a primary energy source for ATP generation. This is further reinforced by the simultaneous up-regulation of carbohydrate hydrolysis and down-regulation of carbohydrate synthesis pathways in addition to a nearly 2-fold increase in lactate dehydrogenase (LDH, 1.8-fold). Similar shifts in apparent glycolytic substrate preference have been noted for a number of other Antarctic species via changes in enzyme activity for LDH, citrate synthase (CS), cytochrome-c oxidase (cyt-c) and hydroxyacyl-CoA dehydrogenase (HOAD). In specimens of a closely related notothenioid, *Pagothenia borchgrevinki*, acclimated to elevated temperature, both LDH and cyt-c activity were found to be elevated [36]. A distantly related species, the eelpout *Pachycara brachycephalum*, exhibited increased cyt-c activity coupled with decreased CS activity at elevated temperatures [37]. Lastly, Jayasundara et al. reported a similar increase in LDH activity coupled with decreased CS activities in *T. bernacchii* specimens acclimated to +4.5 °C [38].

### Cellular death and proliferation

**Fig. 6:**
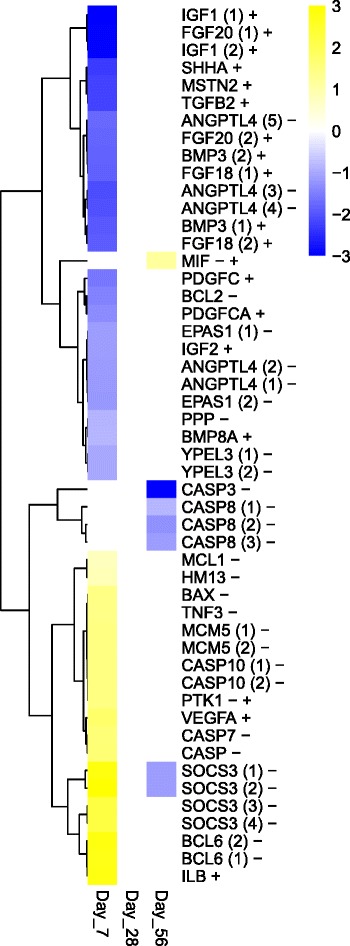
Heatmap apoptosis and cell proliferation: this heatmap demonstrates the change in expression patterns of genes involved in cell proliferation or programmed cell death that displayed differential expression in at least one acclimation time point (7d, 28d, or 56d) when fish acclimated to the multi-stressor treatment (*n* = 4) were compared to fish in the control treatments (*n* = 4). Genes associated with the gene ontology term cell death (GO:0008219) are represented by (−) and cell proliferation (GO:0008283) are represented by (+). Fold change is log_2_ adjusted with genes showing significant up-regulation depicted in *yellow* and genes showing a significant down-regulation in *blue* (FDR ≤ 0.05). Genes whose level of expression did not differ significantly between control and multi-stressor treatments are shown in *white*. The number of gene variants identified in the annotated transcriptome that were associated with the same gene name are indicated in *parentheses* next the gene symbol

In addition to the metabolic changes that were seen in the gill tissue of 7d multi-stressor acclimated fish, we also observed a significant change in the regulation of cell survival with the activation of several genes associated with apoptosis (Fig. 6, Additional file 4: Table S4). Gill tissue isolated from these fish demonstrated a significant up-regulation of a number of caspases (1.7 to 3.0-fold); and multiple suppressors of cytokine signaling (4.5 to 7.4-fold). Interestingly, a down regulation of angiopoietin-related proteins (−2.3 to 4.2-fold,) and endothelial pas domain-containing proteins (−2.2 to −2.3-fold), which can activate the cell’s hypoxia response, are also observed at 7d.

As to cell proliferation, fish in the 7d multi-stressor treatment demonstrated a strong trend of down-regulation overall (4 up-regulated, 20 down-regulated) (Fig. 6, Additional file 5: Table S5) that suggests cell cycle arrest in response to stress extends beyond the initial time-frame proposed by Sleadd and colleagues [39] . Specifically, we observed a strong down-regulation in a number of growth factors including insulin growth factors (−7.7 to −13.0-fold); fibroblast growth factors (−3.6 to −12.1-fold); myostatin (−4.6-fold); bone morphogenic protein (−3.4 to −3.6-fold), and platelet-derived growth factor (−3.0-fold), among others.

### Response to stress

**Fig. 7:**
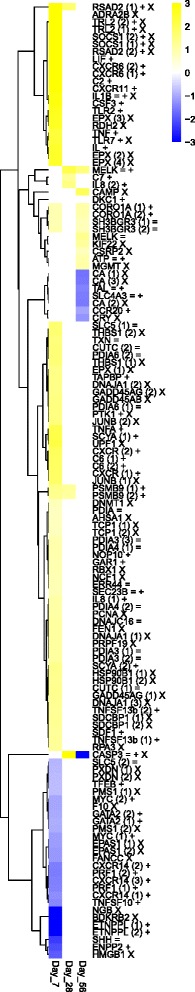
Heatmap–cellular stress response: this heatmap demonstrates the change in expression patterns of genes involved in cellular stress response and homeostasis that displayed differential expression in at least one acclimation time point (7d, 28d, or 56d) when fish acclimated to the multi-stressor treatment (*n* = 4) were compared to fish in the control treatments (*n* = 4). Genes associated with the gene ontology term response to stress (GO:0006950) are represented by (x), immune system process (GO:0002376) are represented by (+), and homeostatic process (GO:0042592) are represented by (=) that displayed differential expression (FDR ≤ 0.05) in at least one time point are included. Fold change is log_2_ adjusted with genes showing significant up-regulation depicted in *yellow* and genes showing a significant down-regulation in *blue*. Genes whose level of expression did not differ significantly between control and multi-stressor treatments are shown in *white*. The number of gene variants identified in the annotated transcriptome that were associated with the same gene name are indicated in *parentheses* next the gene symbol

Our results support previous studies, which found a lack of inducible heat shock response in *T. bernacchii* [26, 40, 41]. Despite the considerable transcriptome-wide changes in expression demonstrated above; of the 64 heat shock and heat shock-related genes expressed in the reference transcriptome, only 7 were found to be differentially regulated (FDR ≤ 0.05) in fish exposed to the 7d multi-stressor treatment (Fig. 7, Additional file 6: Table S6). Overall, of the three HSP families typically induced in a teleost heat shock response [42], there is almost no response for the HSP90, HSP70 and small HSP families. Among those heat shock proteins that did show significant changes in regulation, HSP40 demonstrated the clearest up-regulation with both HSP40 genes found to be up-regulated (2.0 and 1.9-fold) in the 7d multi-stressor treatment group. A similar trend was observed in *P. borchgrevinki* acclimated to ^+^4 °C for 4 days. In this study, Bilyk and Cheng report HSP47 as the only HSP transcript displaying significant up-regulation in this closely related species [25].

While none of the inducible HSP isoforms were differentially regulated, there did appear to be a number of endoplasmic reticulum (ER) specialized molecular chaperones that did show moderate increases in the 7d multi-stressor treatment. For instance, of the over 20 HSP70-related genes annotated in our reference library, only 2 transcripts (*hsp70-12b*, 2.3-fold and *hsp70-14*, 1.9-fold) demonstrated significant up-regulation (2.3-fold and 1.9-fold, respectively). Similarly, GRP94, a member of the Hsp90 family that plays a role in assembly of secreted proteins and is localized in the ER, was up-regulated ~ 2.5-fold in the 7d fish.

In opposition to the lack of a robust HSR, *T. bernacchii* does display a strong response to DNA damage. The gill tissue from fish acclimated to the multi-stressor treatment for 7d displayed up-regulation of a number of genes associated with activation of DNA damage response pathways (Fig. 7, Additional file 7: Table S7). Among the genes up-regulated are several members of the GADD45 family of proteins which mediate the activation of the p38/JNK pathway, resulting in cell cycle arrest, DNA repair, cell senescence, and apoptosis [43]. Furthermore, two key genes directly involved in DNA repair also displayed significant increases. Proliferating cell nuclear antigen (PCNA), which has a stimulatory effect on the 3′-5′ exonuclease activity of DNA polymerase and RAD2, which is a structure-specific 5′-flap endonuclease, were both up-regulated over 2-fold in the 7d multi-stressor acclimated fish.

In one of the few studies that has looked at cell proliferation and DNA damage in Antarctic fish under conditions of cellular stress, Sleadd et al. found a significant increase in PCNA protein concentrations in *T. bernacchii* after being held at ^+^4 °C for 72 h [39]. Unlike our findings at the transcript level, Sleadd et al. found that PCNA protein levels had returned to control values by 168 h (7d). In our multi-stressor study, we found PCNA transcript levels were still significantly elevated in the gill tissue of *T. bernacchii* after a 7d acclimation that included a ^+^4 °C thermal stress. The elevation in this DNA repair machinery tracks the molecular signatures of cell-cycle arrest described above. It is possible the additional stressor (elevated *p*CO_2_) extended the response of PCNA and cell cycle arrest in our study; however, given the differences in tissues observed between the two studies (gill vs liver) and the molecule considered (mRNA vs protein) it is difficult to draw more concrete conclusions.

### Immune system processes

An aggressive immune response is a common characteristic of teleosts when stressed from any number of cellular perturbations [44] and this appeared to hold true for *T. bernacchii*. Genes associated with immune system processes demonstrated a general trend of up-regulation in the 7d multi-stressor treatment (39 up-regulated, 14 down-regulated) (Fig. 7, Additional file 8: Table S8).

This response is highlighted by the up-regulation of a number of chemokines (CXCL6, 11.3-fold; CXCL, 3.7-fold; CXCL11, 14.4-fold) that recruit elements of the immune system including macrophages, t-cells and neutrophils [45, 46]. The notable exception is the down-regulation of CXCL-14 (−2.8-fold), which is thought to inhibit the signaling action of CXCL-12 and immune cell migration [47]. In addition to members of the CXC-type chemokine family, the strong up-regulation of SOCS1, which is thought to serve a vital role in both innate and adaptive immune responses [48, 49], further supports the occurrence of a robust immune response to the multi-stressor treatment at the 7d time-point.

### Homeostatic processes

In teleost fish, the maintenance of ionic and acid–base homeostasis are invariably linked; however, unlike terrestrial organisms, which can alter plasma pH by increasing or decreasing ventilation rates, acid–base balance in fish requires direct exchange of ions with the external environment via specialized chloride cells within the gill epithelium [50]. As such, when experiencing hypercapnic conditions, fish cannot “off-gas” CO_2_ by increasing ventilation rates. The compensation for imbalance requires the direct transfer of an acid (H^+^) and base (HCO_3_^−^) for Na^+^ and Cl^−^ respectively, across the gills, kidneys and/or intestine [51–54].

We found fish acclimated to the 7d multi-stressor treatment demonstrated a robust gene response associated with maintaining acid–base homeostasis. In all, 18 genes in this GO category were significantly up-regulated while only 2 were significantly down-regulated (Fig. 7, Additional file 9: Table S9).

Sodium-hydrogen exchangers are known to be a primary mechanism for acid–base regulation in the gill tissue of fish, with fish excreting protons in order to achieve acid–base homeostasis when subjected to increased CO_2_ concentrations [55]. The acidification resulting from the increased *p*CO_2_ is likely responsible for the observed up-regulation of SLC9A5 (3.2-fold), which is a member of the sodium-hydrogen exchanger family [56]. We do observe a concurrent down-regulation of SLC9A6 (−1.8-fold), however this variant is thought to localize to the mitochondria [57] and not the cellular plasma membrane where SLC9A5 is found, and thus is likely not involved in maintaining cellular pH counter to the external environment.

In addition to perturbations to acid–base balance, increases in temperature and decreases in pH can also impact redox potential within the cell, leading to increased oxidative stress [58]. A common biomarker of oxidative stress, protein carbonyl concentrations, have previously been shown to significantly elevate in *T. bernacchii* exposed to the same multi-stressor conditions [30]. Similarly, in the expression profiles of fish in the 7d multi-stressor treatment, we find the up-regulation of redox related proteins including TXN (thioredoxin, 2.9-fold) and SH3BGRL3 (SH3 Domain Binding Glutamate-Rich Protein Like 3, 1.9-fold).

Interestingly, a number of genes encoding for disulfide-isomerases are also up-regulated (8 gene variants up-regulated at least 1.5 fold) in the 7d multi-stressor treatment. Protein disulfide-isomerases (“PDIs”) are known to assist in proper protein folding or corrective protein folding in the ER and play key roles in the unfolded protein response (UPR) in the lumen of the ER [59, 60]. PDIs are essential in disulfide bond formation and are likely involved in the biogenesis of a large number of membrane bound proteins necessary during cellular remodeling [61]. Their up-regulation is a further indication of the large-scale changes involving membrane bound processes such as ion regulation, acid–base balance and potentially even the redox potential of mitochondria.

## Pathway specific responses (28d and 56d multi-stressor treatments)

### Carbohydrate and lipid metabolism

mRNA expression levels for genes associated with the GO category for carbohydrate metabolism in fish acclimated for longer time-frames displayed distinct changes from the patterns observed in the 7d acclimated fish (Fig. 5, Additional file 2: Table S2). Analysis of significant changes in gene expression for fish in the 28d treatment groups displayed little difference between control and multi-stressor treatments while fish acclimated to the 56d multi-stressor treatment indicated a particular up-regulation of pathways involved in the catabolism of simple sugars (triosephosphate isomerase, 2.1-fold; phosphoglycerate, 2.4-fold; fructose-bisphosphate aldolase, 108.4-fold; phosphomannomutase, 1.8-fold) and the glycolytic enzyme 2-phospho-d-glycerate hydro-lyase (aka enolase, 98.6-fold). Similarly, the number of DE genes involved in lipid metabolism fell to 4 up-regulated/ 0 down-regulated in the 28d fish and 6 up-regulated/ 2 down-regulated in fish acclimated for 56d (Fig. 5, Additional file 3: Table S3).

Following the initial cellular stress response at the 7d time-point, expression of genes associated with metabolic pathways largely appeared to return to basal levels as there were very few differentially expressed genes in fish acclimated to the 28d and 56d multi-stressor treatments. However, the few genes that remain differentially expressed in the longer time periods of 28d and 56d may be evidence of the more persistent metabolic changes that are required for acclimation to elevated seawater temperatures and *p*CO_2_ levels. For example, the 56d multi-stressor treatment experiences a strong up-regulation of multiple diacylglycerol kinase isoforms (2.4-fold, 3.1-fold), a potential indicator of a persistently elevated challenge in maintaining the integrity of cellular membranes. Furthermore, the heavier reliance on carbohydrate utilization and anaerobic glycolysis for ATP generation may also persist beyond the initial cellular stress response observed in the first 7 days. Indeed, this apparent change in energy usage has been shown to persist for at least 14 days in *T. bernacchii* when thermally stressed [38]. Furthermore, we have recently collected LDH activity data in these same fish that when combined with the changes in carbohydrate catabolism pathways noted above, suggests these fish may not be capable of fully compensating for the increased energetic demands of acclimating to this multi-stressor treatment [34].

### Cellular death and proliferation

As seen with lipid and carbohydrate metabolism, the changes in genes associated with cellular death are also heavily diminished in the 28d and 56d acclimated fish (Fig. 6, Additional file 4: Table S4). At 28d few genes are differentially regulated with the notable exceptions of the down-regulation of caspase-8 related genes (−2.0 to −2.7-fold) which continued through the 56d treatment (−2.2 to −2.5-fold decrease). The down regulation of caspase-8 seen in the gill tissue is likely a strong indicator of a reversal of the apoptotic signal induced in the 7d treatment and likely signals a return to normal cell proliferation by 28 days. Caspases are grouped into two functional families, effectors and initiators, of which caspase-8 belongs to the latter [62]. Caspase-8 is known to play a central role in both the cell surface-initiated apoptosis pathway as well as the mitochondrial-initiated apoptosis pathway and its activation is likely a key regulatory step in the initiation of the apoptosis signaling pathway [63, 64]. Fish in both the 28d and 56d multi-stressor groups appeared to exhibit dramatic changes in expression of caspase-3 precursor (+621.7-fold and −3396.9-fold, respectively). However, a closer analysis at the individual level of the expression of this particular gene indicates that these dramatic changes in expression are driven by a single individual within each treatment group, and thus are likely an aberration. The strong negative regulation of the cell proliferation also appears to be limited to the initial cellular stress response as no genes in this GO category were differentially regulated in the fish acclimated for 28d and only one was observed in fish acclimated for 56d in the multi-stressor treatment (Fig. 7, Additional file 5: Table S5).

Our data indicate the positive regulation of cell-cycle arrest and apoptosis observed under acute thermal challenges continue for at least 7 days into a cellular stress event. Taken together with the remodeling of metabolic pathways discussed above and the physiological and biochemical analyses previously performed on these same specimens [30, 33, 34], it appears *T. bernacchii* requires somewhere between 14 and 28 days to transition from a bio-energetically costly cellular stress response to a state of relative cellular homeostasis. However, even after 56 days of continual exposure to a multi-stressor scenario, *T. bernacchii* may not be capable of fully compensating for elevated temperatures and *p*CO_2_ levels.

### Response to stress

As seen in the 7d acclimated fish, we found little evidence of the *T. bernacchii* is capable of mounting an effective heat shock response in gill tissue after chronic exposure to a stressful environment. In the fish acclimated to the 28d multi-stressor treatment we observed zero DE genes in this GO category and only 3 in fish acclimated to the multi-stressor treatment for 56d (Fig. 7, Additional file 6: Table S6). We did however note a conspicuous −4.5-fold decrease in transcript levels for the constitutively expressed chaperone, HSC71 after 56d of acclimation to ^+^4 °C and 1000 μATM *p*CO_2_ (Fig. 7, Additional file 6: Table S6). These data are consistent with results obtained from a previous description of the *de novo* transcriptome assembly of *T. bernacchii* in which we noted a down-regulation of constitutively expressed chaperones in a number of tissues after a 28d acclimation to ^+^4 °C alone [26]. Bilyk and Cheng confirmed a similar trend in liver tissues of *P. borchgrevinki* after a short-term acclimation (2–4 days) to elevated temperatures [25].

These changes in constitutive chaperones also coincide with reduced levels of oxidative damage in the gill tissue of both *T. bernacchii* and *P. borchgrevinki* [30]. Taken together, these unexpected shifts in chaperoning capacity and protein damage suggest acclimation to elevated temperature may have interesting and perhaps unexpected consequences for protein dynamics in these fish.

### Immune system processes

Unlike many of the other GO sub-categories assessed in this study, a more sustained differential gene expression response was observed within immune system processes (7 up-regulated at 28d; 5 up-regulated, 4-down regulated at 56d) (Fig. 7, Additional file 8: Table S8). While it may be possible that the immune system is responding to a latent infection that is able to manifest due to the effects of the multi-stressor treatment on *T. bernacchii*, the more likely scenario is that the observed response is to wide spread cellular damage and remodeling directly resulting from the multi-stress treatment.

This argument is supported by indicators of cellular damage, such as PSMB9, which is also up-regulated at the 7d (2.5-fold) and 28d (2.3-fold) time-points. PSMB9, also known as 20S proteasome subunit β-1i, is an essential subunit of the proteasome and may be associated with a more specialized form of the proteasome known as the immune proteasome [65]. PSMB9 is required for the production of MHC class-1 restricted T-cell epitopes, and thus plays an important role in antigen processing. Furthermore, a previous study measuring stress at the cellular level found that several notothenioids exhibit considerable increases in oxidative damage after 7 days of heat and *p*CO_2_ stress, which diminishes at 28 and 56 days [30]; indicating a sufficient level of protein damage likely exists to result in the activation of the immune proteasome seen here.

### Homeostatic processes

The concurrent up-regulation of pH regulators, redox regulators and disulfide isomerases observed at the 7 day time-point in the multi-stressor condition is indicative of the challenges *T. bernacchii* faces in maintaining homeostasis under these conditions. After the short-term acclimation response, *T. bernacchii* no longer experiences dramatic changes in expression of proteins related to the maintenance of cellular homeostasis. As seen with previous categories, this response tapered off rapidly at 28d with only 2 up-regulated genes, suggesting these fish are capable of at least partially compensating for the changes in extracellular pH and osmolarity likely caused by the multi-stressor condition (Fig. 7). A notable exception is the continued up-regulation of SH3BGRL3 throughout the 56d time point (Fig. 7), which may indicate the continued need for assisted folding of membrane associated proteins under the multi-stressor condition and may be representative of a protracted cost to cellular maintenance. Despite the return of most gene expression patterns to a basal state observed in control fish, the continued up-regulation of some key cellular functions provides further support that *T. bernacchii* may not be fully capable of compensating for these experimental conditions within the 56d acclimation period studied here.

## Conclusion

Our efforts have uncovered several key findings concerning the molecular response of *T. bernacchii* under the multi-stressor condition brought on by increased sea surface temperature and ocean acidification. First, although previous studies using acute thermal stress suggest a portion of the conserved cellular stress response is retained in these fish, we have now shown that *T. bernacchii* is capable of mounting a robust and coordinated response to stress and that this response persists for at least 7 days and perhaps as long as 2 weeks. Among the conserved responses observed were DNA repair pathways accompanied by cell-cycle arrest and apoptosis; maintenance of acid–base balance and redox-potential; and cytokine signaling and cellular inflammation. As found in previous studies under heat stress alone, *T. bernacchii* does not possess an inducible heat shock response when exposed to synergistic heat and *p*CO_2_ stress. However, the up-regulation of chaperones and PDIs localized in the ER may indicate the unfolded-protein response of the ER remains intact in these fish.

Second, the multi-stressor treatment results in significant cellular damage that continues for at least 7 days after the initial exposure. In addition to the increase in oxidatively damaged proteins previously noted in these fish, there also appears to be a significant amount of DNA damage accrued as indicated by the up-regulation of pathways associated with cellular death, DNA damage responses, and immune system responses. Furthermore, the multi-stressor treatment induces a strong initial response that likely comes at a significant energetic cost to the organism. In addition to the activation of stress responsive pathways, we also observed changes in gene expression patterns that suggest a shift in substrate preference for glycolysis and the possible reliance on anaerobic pathways to supplement ATP generation.

Our findings demonstrate that the multi-stressor condition induces a strong short-term response that returns to near basal levels of expression within most of the studied pathways, indicating some degree of compensation to the environmental changes has set in. This would seem to indicate that *T. bernacchii* possesses the physiological plasticity to cope with an environment similar to the multi-stressor condition and thus also with the changing climate of the Southern Ocean.

**Fig. 8:**
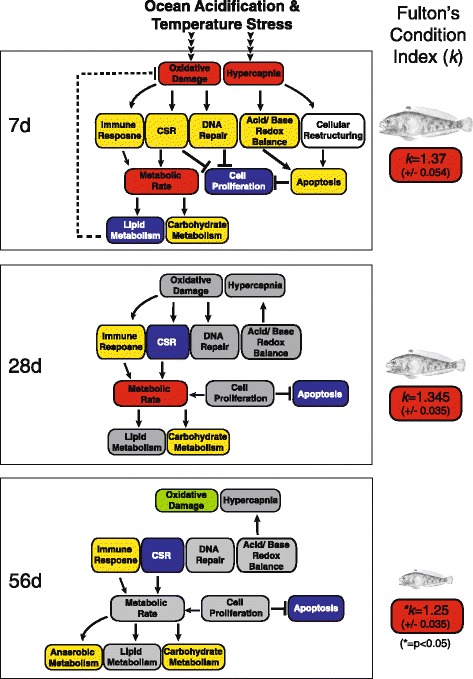
Schematic of molecular response integrated with physiological changes at the organismal level: Gene expression patterns identified in this study suggest a number of cellular pathways that were up-regulated (*yellow boxes*) or down-regulated (*blue boxes*) in fish acclimated to combined elevated temperature and *p*CO_2_ stressors over the course of 56 days. These changes likely underlie the physiological changes previously observed in *T. bernacchii* under these multi-stressor conditions (*red boxes* [30, 33, 34]). Although the expression of most genes returned to basal levels, a number of key genes remained up-regulated at the 56 day time-point suggesting *T. bernacchii* has not fully compensated. Ultimately, these stressors may impact the fish condition overall (as suggested by Fulton’s condition index [34]) and may have long-term population level impacts such as reduced growth and reproduction

Although our study demonstrates that after 56 days of exposure to the multi-stressor condition, expression levels across the transcriptome largely returned to near basal levels, it is noteworthy that not all gene expression patterns returned to control levels and this may be an indication that *T. bernacchii* has not fully compensated within the 56d acclimation period. We identifed subtle changes in expression that persist through the 56d time-point (such as shifts towards carbohydrate metabolism and continued maintenance of membrane protein homeostasis) that may significantly affect *T. bernacchii*’*s* fitness over a longer period of time. Furthermore, in a related study, we tracked growth parameters and calculated Fulton’s index for these fish over the course of the experiment and found temperature had a negative impact on fish condition. In particular, *T. bernacchii* displayed significant declines in condition when acclimated to 4 °C for 28d or longer [34]. As such, we must approach conclusions about their capacity to adapt to future ocean conditions with caution, as this physiological plasticity does not appear to rise to the level of warm hardiness previously observed in other notothenioid species [36]. Figure 8 provides a summary schematic of the molecular responses observed over the 56 day acclimation period and how they may scale up to impact the physiology of these fish in a high CO_2_ ocean.

Lastly, as this study focused upon the gills of adult fish only, and did not investigate juveniles, other tissues, or the overall reproductive cycle; it is difficult to surmise the overall effect that these synergistic stressors may have on the fitness of *T. bernacchii* at the population level and over the course of many generations. Further inquiry is necessary to address these concerns, and to further elucidate *T. bernacchii’s* potential to adapt to changing environmental conditions.

## Methods

### Collection of fish

Specimens of *T. bernacchii* were collected in McMurdo Sound, Antarctica from September through December, 2012. Fish were caught using hook and line through 10-in. holes drilled through the sea ice and transported back to McMurdo Station in aerated coolers where they were housed in flow-through aquaria maintained at ambient seawater temperature (−1.5 °C). Fish were then tank-acclimated under ambient conditions for one week prior to being placed in experimental tanks. All procedures were conducted in accordance with the Animal Welfare Act and were approved by the University of South Carolina Institutional Animal Care and Use Committee (ACUP protocol # 100377).

### Experimental design

We used four, 1240 L experimental tanks to assess the combined effects of elevated temperature and *p*CO_2_ on *T. bernacchii*. Our two experimental treatments consisted of a control tank which was held near ambient conditions (−1 °C and 430 μatm) and a high temperature + high *p*CO_2_ treatment (+4 °C/ 1000 μatm). Fish were placed in experimental tanks and acclimated for a total of 56 days. Five fish per treatment were removed at 7d, 28d, and 56d time-points, after which fish were sacrificed and gill tissues were collected and immediately flash-frozen in liquid nitrogen. Although we recognize a fully replicated experimental design is ideal to exclude tank effects as a possible confounding factor, the constraints of working in Antarctica prevented us from using this approach. However, our previous analyses show no tank effect when treatments were alternated between tanks across multiple seasons [30, 33].

### Manipulation of seawater conditions

Temperature and *p*CO_2_ levels were manipulated within the experimental treatment tanks using a *p*CO_2_ generation system first described by Fangue et al. [66] and adapted for use with large-scale applications and combined with thermostated titanium heaters (Process Technology, Brookfield CT, USA; Enzor et al. [33]). Atmospheric air was pumped through drying columns (filled with drierite) to remove moisture, and air was scrubbed of CO_2_ using columns filled with Sodasorb. Pure CO_2_ and CO_2_-free air were then blended using digital mass flow controllers and bubbled into header tanks that were continuously replenished with ambient seawater using venturri injectors, which in turn fed into experimental treatment tanks.

Temperature, pH (total scale), salinity, total alkalinity (T_A_) and oxygen saturation were measured daily from both incoming seawater as well as experimental treatment tanks. For *p*CO_2_ analysis, we followed the SOP as described in the Best Practices Guide [67] for the spectrophotometric determination of pH using m-cresol purple and measurement of total alkalinity via acid titration using a computer-controlled T50 Titrator (Mettler Toledo, Columbus, OH, USA) . Temperature was measured with a calibrated digital thermocouple (Omega Engineering Inc., Stamford, CT, USA) and salinity was measured using a YSI 3100 Conductivity meter (Yellow Springs, OH, USA). CO_2_*calc* [68], using the constants of Mehrbach et al. [69] as refit by Dickson and Millero [70], was used to calculate all other carbonate parameters. Oxygen saturation was recorded using a galvanic oxygen probe (Loligo Systems, Denmark). Mean values (± s.d.) of temperature (°C) and *p*CO_2_ (μatm) over the course of the experiment were first reported by Enzor and Place [30]. Additionally, treatment tanks were sampled daily for the presence of ammonia, nitrite and nitrates, with no significant increase in waste products noted over the course of the experiment (data not shown).

### Tissue collection and RNA extraction

To obtain individual gill-specific expression profiles, we separately indexed and sequenced RNA samples from gill tissue that had been collected from fish acclimated to the two experimental treatments described above (*n* = 5 fish per treatment) for 7, 28 and 56 days. Immediately after euthanizing the fish, tissues were excised in a −2 °C environmental chamber, flash frozen in liquid nitrogen, and shipped back to our home institution on dry ice where they were stored at −80 °C until used. Total RNA from approximately 100 mg of frozen tissue was extracted using TRIzol (Invitrogen) following the manufacturer’s recommendations. The RNA was further cleaned by re-suspending in 0.1 ml of RNase/ DNase-free water and adding 0.3 ml of 6 M guanidine HCl and 0.2 ml of 100 % ethylalcohol (EtOH). The entire volume was loaded onto a spin column (Ambion) and centrifuged for 1 min at 12,000 × g at 4 °C. Flow-through was discarded, and filters were washed twice with 0.2 ml 80 % EtOH. RNA was eluted off of the filters twice with 0.1 ml of DEPC-treated water. RNA was precipitated by the addition of 0.1 vol of 3 M sodium acetate (pH 5.0) and 2.5 vol of 100 % EtOH, mixed by inversion of tubes and placed at −80 °C for 1 h. After this period, tubes were centrifuged at 12,000 × g for 20 min at 4 ° C. Pellets were washed twice with 80 % EtOH and re-suspended in 30 μl of RNase/ DNase-free water. Lastly, RNA was DNase treated at 30 °C for 10 min. Total RNA from *n* = 5 fish within an acclimation treatment was submitted to the Vaccine and Gene Therapy Institute (VGTI) Florida for quality assessment and determination of specific concentration using an Agilent 2100 BioAnalyzer. From the original samples, the 4 highest quality replicates from each treatment and time point were selected for cluster generation using the Illumina® TruSeq RNA Sample Prep v2 Hs Protocol and sequencing via an Illumina® HiSeq 2500 Rapid Run initialized for single-end 100 bp reads.

### Sequencing read quality control and mapping

Raw reads from each of the 24 samples were processed using Trimmomatic [27]. Illumina® TruSeq RNA Sample Prep v2 HS adapters were removed as well as any bases on the end of the reads with a PHRED33 score of <20 or any portion of the read that did not average at least a PHRED33 score >20 across a minimum span of 4 bp (Trimmomatic parameter input: ILLUMINACLIP:Trimmomatic-0.32/adapters/TruSeq2-SEMultiplex.fa:2:30:10 LEADING:20 TRAILING:20 SLIDINGWINDOW:4:20 MINLEN:75). Following trimming, only sequencing reads ≥75 bp in length were retained. The remaining sequencing reads were individually mapped to an existing transcriptome using Bowtie2-2.2.3 with standard parameters [28]. The transcriptome employed as a reference was an enhancement of the previously published *T. bernacchii* transcriptome [26] using 150 bp paired-end Illumina reads as additional input into the Trinity Assembly program [71] under default parameters. Assembly was conducted on the XSEDE Blacklight Supercomputing Resource utilizing the native Trinity module [72]. The additional Illumina reads were obtained from pooled RNA isolated from the gill, liver and brain tissue of *T. bernacchii*. After assembly the transcriptome was pruned using RSEM [73] with any transcripts with expression values less than 0.001 FPKM removed, and then further compacted using CD-HIT-EST [74] at a percent identity of 100 %. Annotation was facilitated by the XSEDE Stampede Supercomputer [75] utilizing the native ncbi-Blast module in a massively parallel structure [76] and then completed on local resources with the BLAST2GO command line utility [77].

### Differential gene expression analysis

Using raw mapping counts from Bowtie2 [28], RNA-Seq by Expectation-Maximization (“RSEM” version 1.2.18) [75] analyses were conducted to generate estimated read-count (count) and fragments per kilobase million (FPKM) counts for each sample at the transcript and gene level. The Trinity pipeline (Trinity version trinityrnaseq_r20140717) [78] was used to aggregate these counts into master matrices for import into the *R* statistical package [79]. Before import, the samples were grouped by time-point and treatment similarity to conduct pairwise analyses of the effect of the multi-stressor treatment over time as compared to the control. Empirical analysis of digital gene expression data in *R* (“edgeR” version 3.4.2) was implemented to conduct differential gene expression analyses [29]; dispersion values were calculated using the replicate groups; and exact tests utilizing a negative binomial distribution with a cutoff false discovery rate of 0.05 were used to identify differentially expressed transcripts and genes.

A sample-level differential expression heat map was generated from the differential gene expression analyses resulting from edgeR using the Trinity pipeline. Within the BLAST2GO graphical interface package [80] Fisher’s Exact tests (FDR ≤ 0.05) were conducted for differentially expressed transcripts of the multi-stressor treatment to identify over-represented gene ontology terms within the up- and down- regulated transcripts within each treatment group in general. Using custom Python scripts annotation and expression data files were combined, and GO categories and genes of interest were extracted for further analysis.

### Availability of supporting data

The raw data were deposited at the NCBI Sequence Read Archive under the Bioproject accession number PRJNA289753.

## Additional files

Additional file 1: Table S1. GO over representation analysis: This analysis table contains the raw data for the identification of GO terms over-represented in the 7d multi-stressor up-regulated, 7d multi-stressor down-regulated, 28d multi-stressor up-regulated, 28 multi-stressor down-regulated, 56 multi-stressor up-regulated, and 56 multi-stressor down-regulated subgroups. The table provides the GO-ID, GO Term, and number of terms found if over-represented in the subgroup (a blank entry indicates a lack of significance for that GO term). The total number of each GO terms found in the reference transcriptome is also included. (XLSX 21 kb) Additional file 2: Table S2. Carbohydrate metabolic process: This table provides all differentially expressed genes exceeding the confidence threshold (FDR ≤ 0.05) that are associated with the GO Term carbohydrate metabolic process (GO:0005975) at 7d, 28d and 56d (*n* = 4). The table includes the gene sequence name from the reference transcriptome, the BLAST result description, the sequence length, the number of BLAST hits to that gene sequence, the minimum e-value of the BLAST hits, the mean similarity, the # of GO terms associated with that sequence, the list of those GO terms, the Enzyme Codes associated with that sequence, InterproScan results associated with that sequence, the log_2_ adjusted fold change in expression, the log_2_ counts per million (CPM) of the reads mapped to that sequence, and the *p*-value and FDR reflecting the confidence in the displayed differential expression of that gene. (XLSX 23 kb) Additional file 3: Table S3. Lipid metabolic process: This table provides all differentially expressed genes exceeding the confidence threshold (FDR ≤ 0.05) that are associated with the GO Term lipid metabolic process (GO:0006629) at 7d, 28d and 56d (*n* = 4). The table includes the gene sequence name from the reference transcriptome, the BLAST result description, the sequence length, the number of BLAST hits to that gene sequence, the minimum e-value of the BLAST hits, the mean similarity, the # of GO terms associated with that sequence, the list of those GO terms, the Enzyme Codes associated with that sequence, InterproScan results associated with that sequence, the log_2_ adjusted fold change in expression, the log_2_ counts per million (CPM) of the reads mapped to that sequence, and the *p*-value and FDR reflecting the confidence in the displayed differential expression of that gene. (XLSX 20 kb) Additional file 4: Table S4. Cell death: This table provides all differentially expressed genes exceeding the confidence threshold (FDR ≤ 0.05) that are associated with the GO Term cell death (GO:0008219) at 7d, 28d and 56d (*n* = 4). The table includes the gene sequence name from the reference transcriptome, the BLAST result description, the sequence length, the number of BLAST hits to that gene sequence, the minimum e-value of the BLAST hits, the mean similarity, the # of GO terms associated with that sequence, the list of those GO terms, the Enzyme Codes associated with that sequence, InterproScan results associated with that sequence, the log_2_ adjusted fold change in expression, the log_2_ counts per million (CPM) of the reads mapped to that sequence, and the *p*-value and FDR reflecting the confidence in the displayed differential expression of that gene. (XLSX 17 kb) Additional file 5: Table S5. Cell proliferation: This table provides all differentially expressed genes exceeding the confidence threshold (FDR ≤ 0.05) that are associated with the GO Term cell proliferation (GO:0008283) at 7d, 28d and 56d (*n* = 4). The table includes the gene sequence name from the reference transcriptome, the BLAST result description, the sequence length, the number of BLAST hits to that gene sequence, the minimum e-value of the BLAST hits, the mean similarity, the # of GO terms associated with that sequence, the list of those GO terms, the Enzyme Codes associated with that sequence, InterproScan results associated with that sequence, the log_2_ adjusted fold change in expression, the log_2_ counts per million (CPM) of the reads mapped to that sequence, and the *p*-value and FDR reflecting the confidence in the displayed differential expression of that gene. (XLSX 14 kb) Additional file 6: Table S6. Heat shock related proteins: This table provides all genes associated with heat shock proteins found within the reference transcriptome regardless of expression/confidence level at 7d, 28d and 56d (*n* = 4). Each time-point sub-table is sorted by FDR (smallest first). The table includes the gene sequence name from the reference transcriptome, the BLAST result description, the sequence length, the number of BLAST hits to that gene sequence, the minimum e-value of the BLAST hits, the mean similarity, the # of GO terms associated with that sequence, the list of those GO terms, the Enzyme Codes associated with that sequence, InterproScan results associated with that sequence, the log_2_ adjusted fold change in expression, the log_2_ counts per million (CPM) of the reads mapped to that sequence, and the *p*-value and FDR reflecting the confidence in the displayed differential expression of that gene. (XLSX 37 kb) Additional file 7: Table S7. Response to stress: This table provides all differentially expressed genes exceeding the confidence threshold (FDR ≤ 0.05) that are associated with the GO Term response to stress (GO:0006950) at 7d, 28d and 56d (*n* = 4). The table includes the gene sequence name from the reference transcriptome, the BLAST result description, the sequence length, the number of BLAST hits to that gene sequence, the minimum e-value of the BLAST hits, the mean similarity, the # of GO terms associated with that sequence, the list of those GO terms, the Enzyme Codes associated with that sequence, InterproScan results associated with that sequence, the log_2_ adjusted fold change in expression, the log_2_ counts per million (CPM) of the reads mapped to that sequence, and the *p*-value and FDR reflecting the confidence in the displayed differential expression of that gene. (XLSX 26 kb) Additional file 8: Table S8. Immune system process: This table provides all differentially expressed genes exceeding the confidence threshold (FDR ≤ 0.05) that are associated with the GO Term immune system process (GO:0002376) at 7d, 28d and 56d (*n* = 4). The table includes the gene sequence name from the reference transcriptome, the BLAST result description, the sequence length, the number of BLAST hits to that gene sequence, the minimum e-value of the BLAST hits, the mean similarity, the # of GO terms associated with that sequence, the list of those GO terms, the Enzyme Codes associated with that sequence, InterproScan results associated with that sequence, the log_2_ adjusted fold change in expression, the log_2_ counts per million (CPM) of the reads mapped to that sequence, and the *p*-value and FDR reflecting the confidence in the displayed differential expression of that gene. (XLSX 23 kb) Additional file 9: Table S9. Homeostatic process: This table provides all differentially expressed genes exceeding the confidence threshold (FDR ≤ 0.05) that are associated with the GO Term homeostatic process (GO:0042592) at 7d, 28d and 56d (*n* = 4). The table includes the gene sequence name from the reference transcriptome, the BLAST result description, the sequence length, the number of BLAST hits to that gene sequence, the minimum e-value of the BLAST hits, the mean similarity, the # of GO terms associated with that sequence, the list of those GO terms, the Enzyme Codes associated with that sequence, InterproScan results associated with that sequence, the log_2_ adjusted fold change in expression, the log_2_ counts per million (CPM) of the reads mapped to that sequence, and the *p*-value and FDR reflecting the confidence in the displayed differential expression of that gene. (XLSX 15 kb)

## Abbreviations

bp: Base pair
CHR: Cellular homeostasis response
CS: Citrate synthase
CSR: Cellular stress response
Cyt-c: cytochtome-c oxidase
ER: Endoplasmic reticulum
FDR: False discovery rate
FPKM: Fragments per kilobase million
GO: Gene Ontology
HOAD: Hydroxyacyl-CoA dehydrogenase
IPCC: Intergovernmental Panel on Climate Change
LDH: Lactate dehydrogenase
*p*CO_2_: Partial pressure carbon dioxide
RNA-Seq: RNA sequencing
RSEM: RNA-Seq by Expectation-Maximization
SST: Sea surface temperature
UPR: Unfolded protein response
